# Tracing the early dispersal of reindeer in southern Sweden: Chronology, habitat, and human interaction (*c*. 12,000–7000 BCE)

**DOI:** 10.1177/09596836261422209

**Published:** 2026-03-11

**Authors:** Markus Fjellström, Peter Jordan, Anders Angerbjörn, Anna-Kaisa Salmi, Kerstin Lidén

**Affiliations:** 1 Department of Archaeology and Ancient History, Lund University, Sweden; 2 Archaeological Research Laboratory, Department of Archaeology and Classical Studies, Stockholm University, Sweden; 3 Department of Zoology, Stockholm University, Sweden; 4 Faculty of Humanities, Oulu University, Finland

**Keywords:** Early Mesolithic, Late Palaeolithic, subfossil reindeer, δ^13^C, δ^15^N, ^14^C

## Abstract

After the wider deglaciation of Northern Europe, pioneer reindeer populations started to move into southern Scandinavia; however, this process is poorly understood. In this paper we aim to reconstruct dispersal processes of reindeer into southern and western Sweden from the Late Palaeolithic through to the Early Mesolithic, when reindeer disappear from the record. Has presence of reindeer in southern Sweden changed over time, were there changes in habitat and was the hunt of reindeer a possible driving factor to their disappearance? We have assembled and analysed a dataset of 220 unburnt reindeer skeletal elements from wetlands, earthen finds and shell middens from southern and western Sweden. Additional ^14^C-analysis have been performed to set the chronological frame. The results demonstrate that reindeer were present in southern and western Sweden from 12,066 to 7079 cal BCE and that the number of reindeer was highest during the Early Holocene. Stable isotope analyses (δ^13^C and δ^15^N), provided information on changes in reindeer habitat. The marked variation in δ^13^C and δ^15^N values suggests that reindeer grazed in different habitats or that the habitat change over time. We suggest that the decrease and final disappearance of reindeer in the Late Palaeolithic/Early Mesolithic was caused by changes in climate and habitat rather than anthropogenically induced.

## Introduction

The transition from the Late Palaeolithic (*c.* 12,000–10,000 cal BCE) to the Early Mesolithic (*c.* 10,000–6800 cal BCE) represents a dynamic period characterised by rapid climatic shifts with changes in distribution of the fauna in rapidly evolving ecosystems in Scandinavia (e.g. Wastegård, 2022). In this study we focus on one species – the reindeer. The deglaciation in northern Europe, starting *c*. 20,000 cal BCE, led to ice-free regions in nowadays northern Germany, Poland, and the Baltic countries by 15,000 cal BCE (Sommer, 2020: 3). The succeeding rapid recession of the Fennoscandian Ice Sheet from *c*. 16,000 cal BCE to 11,600 cal BCE, driven by a warming climate (Sommer, 2020: 3), created dynamic conditions allowing northward dispersal of various species over land bridges into these new areas, including reindeer and their associated predators, for example, humans.

**Figure 1.**
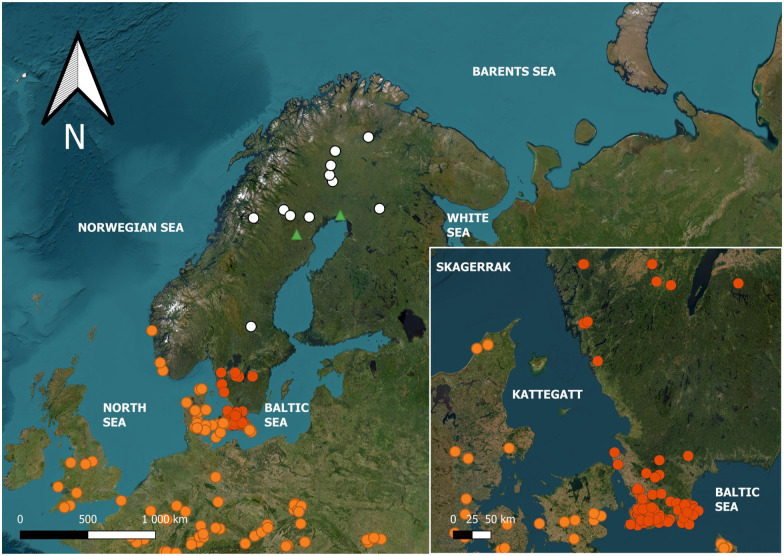
Map overview of reindeer skeletal remains sampled in this study in southern Sweden (red circles) and reindeer from northern Europe and Norway (orange circles; Drucker et al., 2012; Lie, 1990; Sommer, 2020; Sommer et al., 2011). White circles in northern Sweden and Finland represents a selection of cremated reindeer bones found at stone age sites (Bergman et al., 2004; Ekholm, 2024). The green triangles are a selection of two reindeer >30,000 BP (Rankama and Ukkonen, 2001; Fjellström & Lidén, in prep). Map from © QGIS, Esri, with modified background. *Note*. Please refer to the online version of the article to view this figure in color.

The current state of knowledge is that reindeer dispersed over the Danish-Swedish strait during different stages in the Late Palaeolithic and the Early Mesolithic (Björck, 1995; Larsson, 1994), at *c.* 12,000–11,500 cal BCE and *c.* 9200–8800 cal BCE, and from *c.* cal 8250 BCE onwards, after which, they disappear from southern Sweden (Figure 1). Previous ^14^C-analysis of reindeer skeletal remains from southern Sweden demonstrates that reindeer were present on the southernmost tip of the country when the ice started to pull back from the Scandinavian peninsula, already by 13,471–13,872 cal BCE (Larsson, 1994; Magnell et al., 1999; Sommer, 2020; Sommer et al., 2011, 2014)

Reindeer were present in Denmark from *c*. 13,415 to 12,119 cal BCE (our calibration, 12,520 ± 190 BP) to *c*. 8400 cal BCE (Aaris-Sørensen et al., 2007: 915; Holm and Rieck, 1992; Wild et al., 2020), and remains of reindeer from western Norway, indicate presence of reindeer during the Younger Dryas period (Lie, 1990). In central and southern Finland, reindeer finds are scarce from the Late Palaeolithic and Early Mesolithic, with one notable exception of a single antler radiocarbon dated to approximately 32,300 cal BCE (Rankama and Ukkonen, 2001: 133). Other than this, only a limited number of reindeer bones and antlers have been recovered from central and southern Finland. In contrast, around 5750 cal BCE, reindeer are known to have been present in northern Finland (Rankama and Ukkonen, 2001: 133, 136). It has been suggested that mountain reindeer (*Rangifer tarandus tarandus*) dispersed into Finland from northern Norway around 7000 cal BCE (Rankama and Ukkonen, 2001: 139).

In northern Sweden, reindeer remains from Vargforsen have been radiocarbon dated to approximately 31,000–36,000 cal BCE (Fjellström and Lidén, in prep.). Cremated reindeer bones from central and northern Sweden show that reindeer were present from *c*. 8100 BCE (Limsjön 8301 ± 53 BP, 7477–7306 cal BCE, Dumpoktjávratj 8630 ± 85 BP, *c.* 7944–7531 cal BCE, Kangos 8786 ± 59 BP, 8179–7605 cal BCE (Bergman et al., 2004; Wehlin, 2014: 39; Ekholm, 2016)). It has been suggested that the humans (e.g. Ahrensburgians, *c*. 10,700–9000 cal BCE) followed the retreating ice to hunt reindeer and seal. Also, that as the ice regressed reindeer moved into Sweden from the west, that is, Norway (Ekholm, 2024: 61–62).

The reindeer samples analysed in this study have been found as stray finds in southern Sweden during farming activities, or recovered from wetlands, bogs, and kettle holes. Although a few of the skeletal remains have been modified by humans (Magnell et al., 1999), none have been retrieved from an archaeological context. Most of the samples, especially shed antler, are probably derived from living reindeer populations and do not appear to be the result of human predation.

With a focus on geography, chronology and habitat, we aim to investigate the early dispersal of reindeer into, and disappearance from, southern Sweden. Habitat and population density in reindeer are governed by a number of factors (Holand et al., 2022). Environmental changes, such as changes in climate and/or the presence or absence of land bridges over the Danish-Swedish strait, affect reindeer habitat and consequently reindeer population density. Also, hunting and other anthropogenic impact influence population density. We will address the question of the early dispersal of reindeer in southern Sweden by studying (1) chronology in the presence or absence of reindeer in southern Sweden, (2) changes in habitat by analyses of stable δ^13^C and δ^15^N isotopes of the reindeer, and (3) look for evidence of human hunting pressure as a possible factor driving the disappearance of reindeer.

## Major climatic transitions and environmental context

### The Late Pleistocene/Early Holocene transition

The deglaciation of southern Scandinavia (*c*. 18,500 cal BCE and 17,300 cal BCE; see figure 9 in Stroeven et al., 2016: 105), facilitated migration of pioneer species, including many plants and animals, for example, reindeer, as well as humans (Sommer, 2020; Stroeven et al., 2016: 105; Wastegård, 2022: 127). While the Fennoscandian Ice Sheet still covered most of Fennoscandia, northern Germany, Poland, and the Baltic countries were ice free at this time (Sommer, 2020). The Baltic Sea basin was a freshwater lake since there was a land bridge, at the Danish-Swedish strait (Björck et al., 1996; Borzenkova et al., 2015: 33; Larsson, 1994: 172). This land bridge allowed humans, animals and plants to enter into what is now Sweden. Based on a high record of terrestrial insects from southern Sweden, the landscape was dominated by mosses, shrubs and bushes. No wood living insects were recorded, that is, there was an open vegetation of sub-arctic character (Lemdahl, 1988). In western Sweden the early postglacial vegetation was, based on pollen record, dominated by *Betula* and *Hippophae rhaminoides* in low numbers. This vegetation was then slowly replaced by *Pinus* and *Corylus* (Antonsson and Seppä, 2007: 404).

During the Allerød period (*c*. 11,900–10,900 cal BCE), the environment in southern Scandinavia was dominated by an open woodland with birch and stands of pine as well as grass and shrublands, including *Salix* and *Artemisia* (Aaris-Sørensen and Liljegren, 2004: 70; Sommer et al., 2011; see also Wohlfarth et al., 2018). In the southeast, by the coast of the Baltic Sea, *Pinus, Cyperaceae* and *Pocaceae* were the more abundant species (Lagerås et al., 2021: 23–24). Based on the palaeovegetation of Southern Scandinavia, Schenk et al. (2020: 8–9) suggested that summer temperatures in July during the pre-Bølling, Allerød, Younger Dryas and the Preboreal geochronozones were around 16°C. They also suggested that lakes probably were frozen until June (Schenk et al., 2020: 2), which then would allow animals to cross lakes and wetlands until early summer. However, reindeer swim well and could thus cross lakes also during summer if needed (Forbes and Kumpula, 2009: 1361). In Eastern Central Sweden, the Allerød period is marked by high levels of pollen from *Betula, Juniperus, Betula nana, Rubus chamaemorus, Vaccinium* and *Calluna vulgaris* (Blaesild et al., 2024). In Southern Sweden, at the transition into the Younger Dryas, a grass and shrub tundra emerged, favouring reindeer habitats (Björck, 1995: 198, 201). During the Younger Dryas in Eastern-Central Sweden there is an increase in pollen from *Betula, Betula nana, Artemisia* and *Rubus chamaemorus*, and with a low increase of *Pinus* (Blaesild et al., 2024). This is a vegetation that is preferred by reindeer, and indicate a landscape where reindeer would have thrived; however, there are little evidence of their presence during the Late Palaeolithic and Early Mesolithic.

At the beginning of the Preboreal chronozone (*c.* 9750 cal BCE), marking the beginning of the Holocene, the climate becomes warmer and the Fennoscandian Ice Sheet rapidly retreats. In Southern Sweden there is a transformation from open tundra to a park tundra, gradually transitioning into a birch/pine forest, a less favourable habitat for reindeer (Björck et al., 1996: 201; Wastegård, 2022: 127). The Younger Dryas vegetation is replaced by a shrub vegetation mainly dominated by *Juniperus communis* and *Empetrum*. There is also a change in the tree population where *Betula pubescens* and *Populus tremula* colonise southern Scandinavia, with some evidence of *Pinus* (Aaris-Sørensen, 2009: 44; Lagerås et al., 2021: 25). In Eastern Central Sweden, there is an increase of *Artemisia* and *Chenopodiacaea* during the early Preboreal (Björck and Wastegård, 1999), as well as a small increase in trees (*Alnus, Ulmus, Quercus, Tilia* and *Fraxinus)* and shrubs (*Empetrum* and *Calluna vulgaris*; Blaesild et al., 2024). Before the Preboreal oscillation, during the final drainage of the Baltic Ice Lake, sea water flows into the Baltic basin (Borzenkova et al., 2015: 35). The vegetation is now dominated by *Juniperus, Betula* and *Salix* (Björck et al., 2001), suggesting a good habitat for reindeer. However, even though the vegetation was favourable for reindeer, the open water and probably strong currents along western Sweden (Björck, 1995: 28; Jonsson, 2018: 25), would have made it difficult, if not impossible for reindeer to cross. During the latter part of the Preboreal chronozone with a sudden shift to a warmer and more humid climate (Björck et al., 1996) pine increased (Borzenkova et al., 2015: 35)

The Boreal geochronozone that traditionally starts at *c.* 8250 cal BCE is dominated by pollen from *Betula* and *Pinus. Corylus* is also introduced in this period which quickly disperse naturally or anthropogenically in northern Europe. Around 8000 cal BCE, *Corylus, Ulmus, Alnus* and *Quercus* spread across Western Sweden (Antonsson and Seppä, 2007: 404). It is followed by an increase in pollen from *Corylus* alongside a decrease in *Pinus.* However, already at about 8500–8200 cal BCE the vegetation in southern Sweden transitioned into a more deciduous forest with *Corylus* and *Ulmus* (*c.* 7800 cal BCE; Antonsson et al., 2006; Antonsson and Seppä, 2007; Birks, 1994; Björck et al., 2001; Blaesild et al., 2024). Based on a set of ^14^C-dated terrestrial and marine mammals from Southern Scandinavia, Aaris-Sørensen (2009) argued that there is a decline in faunal density in the period between *c.* 9000– and 7000 cal BCE. He connected the decline in abundance of reindeer to rising temperatures, higher precipitation and a mixed and denser deciduous forest (p. 53).

## Postglacial pioneers: Rangifer tarandus

### Reindeer ecology

Reindeer are well adapted to cold climates and have a circumpolar distribution, including the tundra and taiga regions of Fennoscandia, Russia, Siberia and North America (Holand et al., 2022: 6). Whereas reindeer populations in North America are wild, all reindeer in contemporary Sweden are domesticated populations. In Norway, Finland, and North-western Russia there are both domesticated and a few surviving wild reindeer populations. Wild reindeer are also found on Svalbard and were historically introduced into eastern Iceland (Holand et al., 2022: 6, see also figure 3 in Holand et al., 2022). Reindeer are mixed feeders with a diet composed of more than 250 plant species. During summer they feed on a large variation of vascular plants and in winter on lichen, graminoids and moss depending on the altitude and latitude. During wintertime lichen can constitute more than 80% of their diet (Heggberget et al., 2002: 15; Inga, 2008; Ophof et al., 2013). According to Ryg and Jacobsen (1982) and Soppela et al. (2008: 613), lichen provides reindeer with sufficient nutrition during winter, but do not prevent undernutrition. It also feeds on plants higher in protein, such as a variety of grasses, herbs, and shrubs (Klein, 1990; Mårell, 2006: 7; Morris et al., 2018).

Mountain reindeer prefer an alpine and arctic open-tundra, and vegetations such as meadows and heaths. During winter they utilise a broad spectrum of available species (Gaare and Skogland, 1975: 197) and during summer they can afford to be more selective in their diet. For wild mountain reindeer (*Rangifer tarandus tarandus*) from Hardangervidda, *Betula nana* is part of the dung from fall to spring (Gaare and Skogland, 1975: 197–198).

Although, forest reindeer (*Rangifer tarandus fennicus*) might also utilise mountains and tundra, they prefer old lichen-rich forests in winter and wetlands during summer (Kitti et al., 2009; Panchenko et al., 2021).

There are different subspecies of reindeer that exhibit slightly different behaviours. Tundra reindeer is highly gregarious and live in groups of variable sizes that move over large distances, whereas forest reindeer tends to form smaller groups and prefers to remain stationary when conditions are favourable (Holand et al., 2022: 13). Some modern reindeer populations in Alaska and Canada can move more than 5000 km in a year (Galán López et al., 2023), allowing them to cover extensive geographic areas. During winter, reindeer move less to save energy, and during warm summer days they might seek snow patches at higher grounds to cool down and escape insects (see Holand et al., 2022: 14). Drawing parallels with the present-day Yamal region, where reindeer seek windy open plains and coastlines, we have to consider these potential behaviours in Late Palaeolithic and Early Mesolithic reindeer. Reindeer in the tundra would be more secure in a larger group, and tend to gather in large aggregations to escape from predators (Ingold, 1980; Stépanoff, 2017: 389).

Reindeer are unique among cervids, as both males and females possess antlers. Their antlers vary in size and structure between the different subspecies. Where tundra reindeer have long and cylindrical beams, forest reindeer have massive and more palmated antlers. They shed their antlers yearly, so the growth of the antlers provides insights into the nutritional status of the reindeer throughout the year. Adult males cast their antlers after rut in early December, the re-growth starts in early spring. Female and young male reindeer shed their antlers in March–April and the re-growth starts usually directly after shedding (Høymork and Reimers, 2002; see Holand et al., 2022: 8).

### Late Pleistocene/Early Holocene reindeer distribution

During the Late Glacial Bølling/Allerød Interstadial *c.* 12,700–10,900 cal BCE, a warmer climate permitted expansion of trees across Europe, thus altering reindeer distribution (Sommer, 2020: 3, 6). The following transition into the Younger Dryas (*c*. 10,800–9750 cal BCE), led to significant environmental changes, including the decline of reindeer from the European Lowlands. However, reindeer were still present on the European Plain until the Storegga tsunami flooded the coastlines of Scotland, Western Norway and the southern part of the North Atlantic Basin (Nyland et al., 2021), as a *phalanx* of a reindeer, found at the Brown Bank, was ^14^C-dated to 8350 ± 50 BP (GrA-20353, van der Plicht and Kuitems, 2022; 7536–7194 cal BCE, our calibration). Reindeer finds from the Baltic states and Northern Europe suggest that reindeer were present before the tsunami in Finland, Estonia, Lithuania, Latvia, Poland, Germany, Doggerland, England, Scotland, and Ireland (Coard and Chamberlain, 1999; Lie, 1986, 1990; Philippsen et al., 2019; Sommer, 2020; Sommer et al., 2011, 2014; Ukkonen et al., 2006; van der Plicht and Kuitems, 2022; Figure 1). One of the oldest ^14^C-dated reindeer from Scandinavia is from continental Denmark dated to *c.* 12,892–12,233 cal BCE (Wild et al., 2020: 122, 125).

When reindeer first appear in southern Sweden, sometime between 11,623 and 11,146 cal BCE (Magnell et al., 1999), or already by 12,066–11,398 cal BCE according to one ^14^C-date from Börringe (Sommer et al., 2011; Table 1), the environment was favourable for reindeer as it was dominated by *Betula, Salix* and *Artemisia* (Aaris-Sørensen and Liljegren, 2004: 70; Wohlfarth et al., 2018). Larsson (1994) however, suggests there has most likely not been a continuous influx of reindeer into southern Sweden, but rather that reindeer migrations have been disrupted at times for example, when there was no land bridge or no ice. Not only would this have led to a discontinuous migration of reindeer but also that reindeer became isolated on the Swedish side at times (pp. 171–172).

**Table 1.** ^14^C results for this study in BP and cal BCE in 1σ and 2σ.

Landscape	Parish	Site	^14^C-date (BP)	S.d. (BP)	^14^C lab-ID	Cal BCE (1σ)		Cal BCE (2σ)		Cal BCE (2σ) Median	Sigma	Ref
						MAX	MIN	MAX	MIN			
Skåne	Lindby	Hässleberga I	10,128	46	UBA-52570	9882	9671	9986	9455	9793	120	
Skåne	Lindby	Hässleberga I	10,200	130	LuA-4495	10,481	9461	10,523	9402	9938	299	Magnell et al. (1999)
Skåne	Lindby	Hässleberga I	11,390	90	Ua-3296	11,390	11,222	11,499	11,167	11,319	85	Magnell et al. (1999)
Skåne	Lindby	Hässleberga I	10,236	46	UBA-52571	10,042	9877	10,480	9798	9967	120	
Skåne	Lindby	Hässleberga I	10,479	50	UBA-52572	10,667	10,253	10,721	10,154	10,547	149	
Skåne	Lindby	Hässleberga I	9951	48	UBA-52573	9651	9310	9736	9294	9427	114	
Skåne	Lindby	Hässleberga I	11,293	71	UBA-53344	11,291	11,164	11,359	11,146	11,242	62	
Skåne	Lindby	Hässleberga I	10,256	47	UBA-52574	10,092	9881	10,493	9809	10,011	148	
Skåne	Lindby	Hässleberga I	10,920	140	LuA-4491	11,041	10,794	11,161	10,738	10,930	119	Magnell et al. (1999)
Skåne	Lindby	Hässleberga I	11,160	70	UBA-53345	11,215	11,049	11,278	10,947	11,132	80	
Skåne	Lindby	Hässleberga I	9936	45	UBA-52575	9449	9307	9662	9290	9396	103	
Skåne	Lindby	Hässleberga I	9790	75	UBA-50513	9325	9164	9450	8861	9263	120	
Skåne	Lindby	Hässleberga I	10,450	140	LuA-4496	10,667	10,150	10,772	9931	10,364	236	Magnell et al. (1999)
Skåne	Lindby	Hässleberga I	10,867	67	UBA-53346	10,886	10,792	11,012	10,771	10,852	60	
Skåne	Lindby	Hässleberga II	10,450	140	LuA-4496	10,667	10,150	10,772	9931	10,364	236	Magnell et al. (1999)
Skåne	Lindby	Hässleberga II	9701	60	UBA-53347	9261	8934	9291	8844	9166	131	
Skåne	Lindby	Hässleberga II	10,770	150	LuA-4493	10,946	10,561	11,145	10,256	10,794	175	Magnell et al. (1999)
Skåne	Lindby	Hässleberga II	9939	56	UBA-50514	9651	9298	9739	9286	9421	119	
Skåne	Lindby	Hässleberga II	9914	61	UBA-53348	9509	9287	9733	9259	9390	116	
Skåne	Lindby	Hässleberga II	9694	62	UBA-50515	9258	8930	9289	8839	9154	133	
Skåne	Lindby	Hässleberga II	11,185	64	UBA-50516	11,221	11,122	11,283	10,975	11,160	68	
Skåne	Lindby	Hässleberga IV	10,102	46	UBA-52583	9868	9465	9923	9451	9732	126	
Skåne	Lindby	Hässleberga IV	10,592	47	UBA-52584	10,736	10,556	10,769	10,538	10,686	68	
Skåne	Lindby	Hässleberga VII	11,311	48	UBA-52585	11,338	11,175	11,352	11,166	11,256	51	
Skåne	Glemmingebro	Ingelstorp	9784	38	Ua-76715	9287	9247	9308	9222	9265	26	
Skåne	Glemmingebro	Ingelstorp	9353	49	UBA-51863	8706	8552	8761	8462	8618	77	
Skåne	Vellinge	Månstorps torvmosse	9354	48	UBA-52586	8706	8553	8758	8465	8619	75	
Skåne	Ystad	Södra Vallösa mosse	9691	55	UBA-50517	9253	8935	9283	8844	9160	129	
Skåne	Staffanstorp	Brågarp	9840	38	Ua-76716	9312	9263	9371	9245	9292	35	
Skåne	Östra Tommarp	Tomarps torvmosse	9493	57	UBA-51866	9117	8651	9126	8629	8822	150	
Skåne	Lyngby	Lyngby slätter	10,170	120	Lu-3249	10,094	9455	10,511	9374	9865	280	Sommer et al. (2011)
Skåne	Östraby	Hjärsås 3	10,776	68	UBA-51884	10,818	10,750	10,891	10,675	10,791	46	
Skåne	Östra Vemnerlöv	Gyllebo mosse	10,273	55	UBA-50519	10,475	9883	10,510	9867	10,065	175	
Skåne	Mölleberga	Lilla Mölleberga nr 3	9720	62	UBA-53349	9283	8949	9301	8846	9189	128	
Skåne	Villie	Katslösa	10,301	59	UBA-51867	10,488	9986	10,516	9881	10,144	177	
Skåne	Genarp	Häckeberga	10,301	40	Ua-76717	10,479	9990	10,510	9890	10,114	162	
Skåne	Örsjö	Örsjödal	8235	38	Ua-74069	7333	7178	7454	7079	7253	85	
Skåne	Spjutstorp	Spjutstorp	11,552	44	Ua-76718	11,515	11,406	11,545	11,370	11,466	50	Magnell et al. (1999)
Skåne	Svedala	Svedala	10,107	71	UBA-51868	9879	9456	9988	9402	9723	158	
Skåne	Bjäresjö	Gamle mosse	10,921	60	UBA-51869	10,941	10,809	11,105	10,792	10,881	71	
Skåne	Häckeberga	Gyllebo	10,756	52	UBA-52587	10,803	10,766	10,868	10,725	10,783	26	
Skåne	Löderup	Hagesta mosse	10,041	66	UBA-52227	9777	9449	9866	9331	9604	142	
Skåne	Malmö	Husie	9763	57	UBA-50522	9297	9213	9325	8926	9248	89	
Skåne	Lilla Slågarp	Annarp	9746	51	UBA-51870	9290	9200	9307	8929	9237	91	
Skåne	Sjörup	Slimminge	9723	61	UBA-52228	9285	8953	9301	8850	9195	125	
Skåne	Hyby	Vinninge mosse	9931	71	UBA-52229	9653	9293	9745	9264	9432	133	
Skåne	Grönby	Grönby	9701	67	UBA-52230	9266	8929	9294	8835	9156	135	
Skåne	Glostorp	Glostorp	11,496	67	UBA-50521	11,495	11,364	11,547	11,242	11,424	70	
Skåne	Maglarp	Albäckborg	10,918	53	UBA-51871	10,934	10,810	11,036	10,792	10,873	62	
Skåne	Torsjö	Bara	10,524	51	UBA-51872	10,672	10,533	10,742	10,251	10,603	102	
Skåne	Gylle	Annarps mosse nr 2	9849	40	Ua-76719	9320	9261	9442	9247	9299	42	
Skåne	Tolånga	Tolånga socken	9953	47	UBA-51873	9651	9312	9736	9295	9429	114	
Skåne	Bara	Bara	9812	73	UBA-52233	9364	9225	9652	8930	9283	108	
Skåne	Dagstorp	Dagstorp	9890	52	UBA-51874	9443	9286	9655	9251	9351	87	
Skåne	Svedala	Aggarp	9244	47	UBA-52588	8551	8351	8612	8305	8460	80	
Skåne	Sankt Olof	Snapparps mosse	10,230	52	UBA-51875	10,046	9871	10,484	9705	9962	134	
Skåne	Västra Nöbbelöv	Tingaröds mosse	9550	49	UBA-52589	9122	8796	9161	8744	8962	123	
Skåne	Sjörup	Sjörup	9807	70	UBA-52234	9361	9222	9512	8930	9278	100	
Skåne	Skurup	Djurholmen	9899	50	UBA-51876	9443	9291	9656	9256	9358	89	
Skåne	Torup	Bara	10,051	46	UBA-51877	9755	9453	9862	9392	9617	122	
Skåne	Skurup	Saritslöv	9961	71	UBA-52235	9657	9313	9781	9290	9488	138	
Skåne	Tryde	Tryde	10,565	67	UBA-52231	10,735	10,540	10,779	10,256	10,646	106	
Skåne	Arrie	Arrie	10,107	69	UBA-52236	9878	9456	9987	9405	9724	155	
Skåne	Holmby	Holmby	9781	63	UBA-52237	9309	9220	9442	8929	9259	91	Sommer et al. (2011)
Skåne	Skurup	Saritslöv	9617	77	UBA-52238	9208	8844	9246	8778	9003	136	
Skåne	Skivarp	Almaröds mosse	9247	49	UBA-51878	8553	8351	8615	8305	8464	82	
Skåne	Sjörup	Södra Vallösa mosse	8588	65	UBA-52239	7711	7539	7769	7515	7613	72	
Skåne	Bollerup	Bollerup	10,068	40	Ua-76720	9796	9456	9864	9448	9663	119	
Skåne	Skurup	Skurup köping	9548	75	UBA-52240	9125	8773	9218	8656	8950	144	
Skåne	Sjörup	Sjörup	10,003	75	UBA-52241	9739	9371	9858	9306	9552	146	
Skåne	–	Södra Skåne	10,649		UBA-52242	10,781	10,667	10,801	10,534	10,711	81	
Skåne	Skurup	Saritslöv	9818	64	UBA-52243	9326	9232	9451	9151	9286	80	
Skåne	Skurup	Saritslöv	9889	64	UBA-50520	9446	9274	9662	9245	9363	109	
Skåne	Anderslöv	Anderslöv	10,070	49	UBA-51879	9800	9455	9870	9402	9662	127	
Skåne	Kullabygden	Kullabygden i kärrhåla	10,825	40	Ua-76721	10,821	10,781	10,877	10,778	10,806	28	
Skåne	Mölleberga	Lilla Mölleberga 3	9904	46	UBA-52590	9442	9294	9655	9261	9359	84	
Skåne	Dagstorp	Dagstorps mosse	9965	40	LuS-19097	9651	9320	9735	9306	9439	110	
Skåne	Skurup	Hylteberga II	9951	47	UBA-52591	9650	9310	9735	9294	9426	113	
Skåne	Munkarp	Mickelsmossen	11,205	50	UBA-51880	11,212	11,146	11,286	11,060	11,177	40	
Skåne	Munkarp	Mickelsmossen	11,367	62	UBA-50523	11,352	11,231	11,446	11,167	11,296	60	
Skåne	Munkarp	Mickelsmossen	10,696	67	UBA-50524	10,793	10,681	10,808	10,552	10,749	56	
Skåne	Bönarp	Skabersjö	9868	62	UBA-51881	9440	9260	9656	9233	9335	95	
Skåne	Munkarp	Mickelsmossen	10,864	59	UBA-50525	10,879	10,797	10,949	10,779	10,846	50	
Skåne	Södervidinge	Allarps mosse	10,550	50	LuS-19098	10,721	10,541	10,750	10,526	10,635	80	
Skåne	Lindby	Hässleberga 91	11,020	60	LuS-19099	11,112	10,896	11,142	10,833	11,000	80	
Skåne	Gustav	Börringe	9819	68	UBA-52244	9361	9231	9650	8948	9288	92	Sommer et al. (2011)
Skåne	Gustav	Börringe	11,430	130	Lu-3256	11,468	11,230	11,626	11,150	11,362	117	Sommer et al. (2011)
Skåne	Österlen	ÖM:a	10,150	120	Lu-3247	10,021	9454	10,498	9326	9819	272	Sommer et al. (2011)
Skåne	Österlen	ÖM:b	10,000	120	Lu-3250	9739	9367	9856	9304	9548	146	Sommer et al. (2011)
Skåne	Rörum	Rörums mosse	10,140	120	Lu-3253	9995	9454	10,493	9320	9798	267	Sommer et al. (2011)
Skåne	Tranås	Rans mosse	9990	120	Lu-3252	9741	9321	9977	9257	9565	195	Sommer et al. (2011)
Skåne	Arrie	Risebjer	11,500	130	Lu-3265	11,541	11,293	11,652	11,170	11,423	123	Sommer et al. (2011)
Skåne	Arrie	Risebjer	11,220	130	Lu-3255	11,348	11,049	11,446	10,884	11,178	133	Sommer et al. (2011)
Skåne	Arrie	Risebjer	10,120	120	Lu-3260	9985	9454	10,484	9306	9756	256	Sommer et al. (2011)
Skåne	Gustav	Börringe	10,350	120	Lu-3268	10,520	10,003	10,673	9803	10,247	234	Sommer et al. (2011)
Skåne	Gustav	Börringe	11,540	130	Lu-3263	11,628	11,306	11,791	11,214	11,457	128	Sommer et al. (2011)
Skåne	Bönarp	Skabersjö	9964	64	UBA-51882	9656	9316	9759	9292	9484	133	
Skåne	Falsterbo	Falsterbokanalen	9155	53	UBA-51536	8428	8290	8544	8278	8376	72	
Skåne	Falsterbo	Falsterbokanalen	9576	55	UBA-51537	9131	8817	9207	8772	8983	123	
Skåne	Falsterbo	Falsterbokanalen	11,140	61	UBA-51538	11,206	11,024	11,216	10,958	11,107	73	
Skåne	Falsterbo	Falsterbokanalen	9182	50	UBA-51539	8453	8300	8547	8289	8395	73	
Skåne	Falsterbo	Falsterbokanalen	10,752	61	UBA-51540	10,806	10,755	10,874	10,675	10,780	37	
Skåne	Falsterbo	Falsterbokanalen	9435	54	UBA-51541	8777	8631	9116	8557	8719	118	
Skåne	Gladsax	Örnaberga	9870	110	Lu-3266	9655	9236	9857	8941	9389	187	Sommer et al. (2011)
Skåne	Sankt Olof	Kyrkeröd	10,980	130	Lu-3264	11,104	10,820	11,166	10,781	10,969	110	Sommer et al. (2011)
Skåne	Lilla Slågarp	Annarps torvmosse	9742	45	UBA-52592	9286	9207	9299	8941	9238	80	
Skåne	Anderslöv	Anderslöv	9441	58	UBA-51546	8794	8631	9119	8557	8729	131	
Skåne	Sankt Olof	Ekeröd	9805	57	UBA-51545	9307	9241	9443	9162	9276	61	
Skåne	Västerstad	Askeröds mosse	9770	105	St-12968	9367	8927	9655	8809	9228	189	
Skåne	Genarp	Galtasjön	9742	57	UBA-51544	9289	9164	9313	8866	9228	108	Sommer et al. (2011)
Skåne	Glimminge	Bolshög	10,006	54	UBA-51542	9737	9384	9788	9322	9546	127	
Skåne	Simrishamn	Smedstorp	9654	53	UBA-51543	9234	8872	9252	8829	9069	129	
Västergötland	Örgryte	Lilla Torp	10,752	61	UBA-51528	10,806	10,755	10,874	10,675	10,780	37	
Västergötland	Örgryte	Gubberogatan	11,182	66	UBA-51529	11,223	11,063	11,284	10,968	11,157	72	
Västergötland	Hångsdala	Hångsdala	9889	61	UBA-51530	9446	9277	9660	9247	9359	104	
Västergötland	Örgryte	Fräntorp	10,742	70	UBA-51531	10,806	10,740	10,874	10,670	10,774	50	
Västergötland	Marka	S Nyhems gård	8974	51	UBA-51532	8279	8015	8289	7960	8186	100	
Bohuslän	Uddevalla	Kuröd	9566	53	UBA-51533	9125	8813	9196	8759	8977	122	
Bohuslän	Torsby	Bräckebankerna	9523	51	UBA-51535	9119	8751	9142	8651	8908	136	

Reindeer in western Sweden have been dated to *c.* 8300–5700 cal BCE (Boëthius, 2018: 106–107). By the end of the Ice Age reindeer was not present around the area of Skagerrak in southern Norway (Jonsson, 2018: 19). Jonsson claims that reindeer could only be present if the climate permitted winter grazing. Björck et al. (1996: 211) suggest a potential new migration route alongside western Sweden and the Närke strait that could have granted reindeer access to southern Norway.

The youngest radiocarbon dated reindeer in Denmark dates to *c.* 7250 cal BCE whereas the youngest reindeer in southern Sweden is from a bog in Bara dated to between 8560 and 7966 cal BCE (Table 1; Hedges et al., 1997). This is at the end of the Preboreal geochronozone where we see a change in climate and vegetation, with a sudden shift towards a warmer and more humid climate and with an increase of pine pollen (Björck et al., 1996; Borzenkova et al., 2015: 35).

## Postglacial pioneers: People

The pioneer humans in southern Scandinavia arrive in the Late Palaeolithic and are culturally grouped into the Hamburg, Bromme and Ahrensburg cultures ( Andersson and Knarrström, 1999:80; Larsson, 1994: 170; Riede, 2014a). The *Hamburg culture* thrived in the Arctic tundra landscape during the Bølling period, focussing their economy on reindeer hunting (Andersson and Knarrström, 1999: 85). Riede (2014a) suggests that the appearance of the Hamburgian culture in southern Scandinavia coincides with the initial arrival of reindeer in this area (pp. 34–35). Further, that the Hamburgian techno-complex represents a mobile hunting strategy with temporary residential camps (Riede, 2014a: 36). A group of the oldest Hamburg culture, the Havelte group, is believed to have been present in southern Scandinavia, as supported by a few finds from sites in Skåne and Halland (Andersson and Knarrström, 1999; Larsson, 1994: 88). A worked reindeer antler from Slotseng in Denmark (*c.* 10,550 BCE) attributed to the Havelte group, is the oldest evidence of human presence in this region (Aaris-Sørensen et al., 2007: 915–916).

Evidence for early human presence in southern Sweden, here modified reindeer skeletal remains from Hässleberga, have been dated to between 11,623–11,146 and 9923–9320 cal BCE corresponding to the *Bromme* and *Ahrensburg cultures* (Magnell et al., 1999: 10, 17). The Hässleberga kettle hole site has been interpreted as a reindeer kill and processing site connected to activities such as butchering, marrow fracturing and working of antler (Magnell et al., 1999: 17–18). This kettle hole has an osteological assemblage of fish, birds, arctic fox, moose, mountain hare, wild horse, reindeer and one fragment of a left tibia of a human. The wild horse, contemporary to reindeer, exhibits similar kinds of bone modification as reindeer (Magnell et al., 1999: 12). A few of the bones with modification have trampling marks probably made by the melting and retrieving ice, and some has marks from carnivores. Magnell et al. (1999) suggests that the animal skeletal remains found at Hässleberga were probably not deposited directly after the death of the animal (p. 12).

The *Bromme culture* dates to the earliest part of Allerød and has been geographically delimited to northernmost Germany, Denmark and southern Sweden (Riede, 2014b: 36, 71). According to Riede (2014b), the eruption of the Laacher See volcano in northern Germany *c.* 11,000 cal BCE, could have been a trigger for hunter-gatherer communities to partial abandonment of camps and a possible migration Northwards (pp. 68, 71). Also, in the Bromme culture reindeer seems to be an important resource to the economy (Andersson and Knarrström, 1999: 92, 100). In contrast to the Hamburg culture, the Bromme culture is geographically less widespread and has a simpler lithic technology (Andersson and Knarrström, 1999: 103). Notable is that the existence of the Bromme culture in southern Sweden coincides with the establishment of a land bridge between *c.* 9350 and 8950 cal BCE (Larsson, 1994: 172, 174).

From the *Ahrensburg culture* only a few sites are known in southern Scandinavia. Artefacts made of reindeer antlers for example, axes, and harpoons made of different faunal species, are connected to the Ahrensburg culture in the northern European plain (Street et al., 2001). As in the previous culture reindeer were an important part of the economy (Andersson and Knarrström, 1999: 108–110). One of the core organic artefacts connected to the Ahrensburg culture is the Lyngby Axe, also found along the Baltic coast (Girininkas et al., 2016; Philippsen et al., 2019; Ukkonen et al., 2006). The Lyngby Axe, made of reindeer antlers, has been suggested to be used to break the ice for aquatic hunting with harpoons (Cziesla, 2018: 67–68).

Based on the study of lithic material from a few sites in south-eastern Sweden, the Late Palaeolithic is a period with a low human population density where there is no competition in resource management or need for colonisation of new areas (Persson and Knarrström, 2021: 46). The Bro 597 site, dated to *c.* 9500 cal BCE, is to be understood as a site situated in an open landscape that was used for hunting terrestrial and marine mammals, and fishing (Persson and Knarrström, 2021: 80). Interestingly, no reindeer remains have been found at this site.

The slightly later Mesolithic *Hensbacka culture* in western Sweden primarily hunted marine mammals, as evidenced from artefacts such as harpoons (Andersson and Knarrström, 1999:103–104). The earliest phase of Hensbacka culture, dates to 8350–7750 cal BCE (Schmitt et al., 2006: 21). It is a more regional and seasonal culture that later is followed by the *Sandarna culture* (Andersson and Knarrström, 1999: 104; Schmitt et al., 2006).

Further northeast, early human activities at Dagsmosse in Central Sweden are indicated by a find of a red deer without phalanges, radiocarbon dated to 8740 ± 41 BP (our calibration: 7943–7604 cal BCE), and a juvenile elk dated to 8883 ± 35 BP (our calibration: 8232–7853 cal BCE) with skull fractures, both trauma interpreted to have been caused by humans (Blaesild et al., 2024).

Human genetic data suggest that Early Mesolithic humans came into Scandinavia via at least two different routes; from the south and from the northeast following the ice-free Norwegian coast (Günther et al., 2018: 1, 10). If human populations were reindeer hunters and were following reindeer migrating, the mobility of the Early Mesolithic human populations was related to reindeer mobility.

## Material

We located all Late Palaeolithic and Early Mesolithic reindeer material found in southern and western Sweden (Table 1 and Supplemental Table S1, Figure 1). All in all, we analysed 234 samples of unburnt reindeer (antler, bones, teeth), for stable isotope analysis. Of these 234 samples we radiocarbon dated 110 samples. Most of the skeletal remains are finds from different bogs, kettle holes, shell banks and some of them are described as earthen finds.

We have added previously published ^14^C-dated reindeer samples (*n* = 72). One of these is a burnt reindeer bone from a hearth (A50) from Limsjön, north-central Sweden, radiocarbon dated to 7518–7087 cal BCE (8301 ± 59 BP, Ua-46094; Wehlin, 2014: 83). Another is the radiocarbon date of a reindeer antler from Älkärr in eastern Sweden (9319–8760 cal BCE, 9690 ± 105 BP, Ua-2274; Hallgren, 2018; Schött and Isberg, 1931). This antler has not been recovered, thus we were not able to perform isotope analysis on this sample. More problematic is a reindeer antler from Edared in western Sweden that is reported to have been radiocarbon dated to 12,898–11,931 cal BCE (12,265 ± 100 BP, St-2470; Mörner, 1969, see also Nordqvist, 2021: 47); however, Fredén (1984) reports the same date as 11,265 ± 100 BP. If Mörner’s radiocarbon date is right, then the reindeer from Edared is the oldest reindeer dated in western Sweden; however, this date should be taken lightly and unfortunately, the antler from Edared has been impossible to locate.

Different skeletal tissues have different turnover for organic compounds. Bone organic tissue generally remodels over an individual’s lifetime, representing approximately the last 15 years for a large mammal (Hedges et al., 2007); antlers are shed every year, thus reflect the environmental conditions over a period of less than a year (see Holand et al., 2022). Antler starts to grow at the pedicle that is attached to the skull and new tissue is continuously deposited at the tip of the growing antler (see Schwartz-Narbonne et al., 2021: 534). During growth, the antler is supplied with nutrients and oxygen by a covering. Once the antler is finished no further remodelling occurs (Schwartz-Narbonne et al., 2021: 534–535).

## Methods

### Radiocarbon analysis

In order to set the chronological frame of presence and dispersal of reindeer during the Late Pleistocene and Early Holocene in southern and western Sweden dating is crucial. Dating is also important to study changes in reindeer habitat and environment.

Only samples for which the collagen quality fulfilled the standard quality criteria for isotope analyses (Ambrose, 1990; DeNiro, 1985; van Klinken, 1999) were considered for radiocarbon analysis (*n* = 116). The samples were sent for radiocarbon dating at the Ångström Laboratory at Uppsala University, Sweden (Ua-series), the Chrono Centre at Queen’s University in Belfast, Ireland (UBA-series) and the Lund University Radiocarbon Dating Laboratory, Sweden (Lu-series). Calibration of the radiocarbon dates were done using OxCal v.4.4 (Table 1, Supplemental Tables S1 and S2). All calibrated values are given with 2σ. The samples from Hässleberga constitute the largest number of reindeer skeletal remains (*n* = 85). Of these, eight samples were previously radiocarbon dated, and of which two did not fulfil the quality criteria (Magnell et al., 1999; Supplemental Table S1). We have radiocarbon dated another 24 samples from Hässleberga, and 86 samples from different sites in Sweden, in total 110 samples (Table 1 and Supplemental Table S1).

### Stable δ^13^C and δ^15^N isotopes

In order to address how reindeer dispersed into southern and western Sweden in the Late Pleistocene and Early Holocene we address the question of environmental changes and reindeer grazing patterns using δ^13^C and δ^15^N stable isotopes.

The δ^13^C isotope value is determined by the photosynthetic pathway that different plants use, or by the dissolved carbonate in aquatic environments (Sealy, 2001: 270; Van Den, 1982: 596). The δ^15^N values vary due to position in the trophic level in the food chain (with an increase of 3‰–5‰ per trophic level, Bocherens and Drucker, 2003; Minagawa and Wada, 1984), physiology, precipitation, temperature, altitude, or soil composition (Ambrose, 1990; Amundson et al., 2003; Barboza and Parker, 2006; DeNiro and Epstein, 1978; Drucker et al., 2003, 2012; Nieminen and Pietilä, 1999; O’Connell and Hedges, 1999; Parker et al., 2005).

Collagen was extracted from antler, bone, and teeth using a modified Longin method (Brown et al., 1988). For samples that did not fulfil the quality criteria (% of carbon and nitrogen, collagen yield (>1%) and C/N (2.9–3.6; Ambrose, 1990; DeNiro, 1985; van Klinken, 1999), a second extraction was performed now including a 0.1 M NaOH wash in order to remove potential humic acids (Harris, 2020: 48). The collagen was then weighed into tin capsules (between 0.4 and 0.6 mg) and sent for further analysis at Mass Spectrometry Laboratory, Vilnius University, Lithuania, using a Flash EA 1112 Series Elemental Analyzer connected to a Delta V Advantage Isotope Ratio Mass Spectrometer (IRMS) via a ConFlo III Interface with a precision of ±0.1‰ for both δ^13^C and δ^15^N. Isotope ratio data was normalised to IAEA standards: IAEA caffeine (δ^13^C = −27.771, δ^15^N = 1), USGS24 (δ^13^C = −16.05), IAEA-N-1 (δ^15^N = 0.43). All stable isotope values are expressed in the conventional way in permil (‰; Peterson and Fry, 1987).

## Results

### Radiocarbon dating

**Figure 2.**
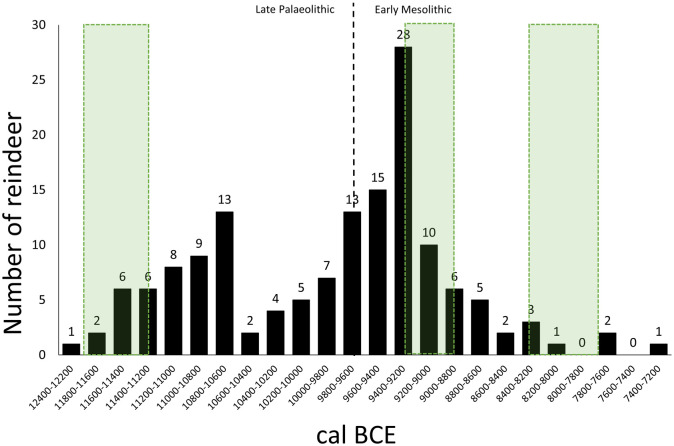
Number of radiocarbon dates of reindeer remains found in southern Sweden, plotted with 200 years cal BCE intervals (e.g. Hedges et al., 1997; Magnell et al., 1999; Sommer et al., 2011). The green boxes are estimated periods of presence of land-bridges between continental Europe and Sweden (Björck, 1995). *Note*. Please refer to the online version of the article to view this figure in color.

In total we radiocarbon dated 110 out of 234 reindeer samples. Dates fall between 11,547 and 11,370 cal BCE (2σ, Ua-74069) and 7454–7079 cal BCE (2σ, Ua-76718; Table 1, Supplemental Tables S1 and S2, Figures 2 and 4a and b), that is, spanning from the very Late Palaeolithic to the Early Mesolithic, suggesting that reindeer were present in southern and western Sweden during cold, temperate as well as warmer climates. Our dates are similar to the previously ^14^C-dated reindeer samples from Skåne (Magnell et al., 1999; Sommer et al., 2011) that we have re-calibrated to 12,066–7966 cal BCE (2σ). However, in this study we demonstrate that reindeer were present in southern Sweden for almost 900 years longer than previously known.

Of all samples, previously dated included, seven reindeer samples from Western Sweden were radiocarbon dated to between 11,157 and 7960 cal BCE, while 141 samples from southern Sweden were radiocarbon dated to 11,547–7079 cal BCE (Table 1 and Supplemental Table S2).

Four reindeer turned out to be historic or modern (Table 1): one reindeer from southern Sweden was radiocarbon dated to 1431–1621 cal CE (413 ± 29 BP, 2σ, Ua-76722) and two reindeer from western Sweden were radiocarbon dated to 1643–1950 cal CE (212 ± 29 BP, 2σ, UBA-51534) and to 1692–1919 cal CE (88 ± 25BP, 2σ, UBA-52245). Reindeer were not naturally present in these regions during this period, hence the reindeer antlers must have been imported or deposited. Another reindeer from western Sweden was radiocarbon dated to 1643–1950 cal CE (245 ± BP, 2σ, LuS-19100). These four reindeer are thus excluded from further discussion.

Previously ^14^C dated reindeer (*n* = 7) that did not meet the requirements for well-preserved collagen (C/N = 2.9–3.6) have not been included in this study. Additionally, two newly ^14^C dated reindeer in this study did not meet the requirement. All those ^14^C results dates have been omitted from the study (Table 1). Also, seven samples of reindeer from Hässleberga were lost during analysis (sample numbers UBA-52576 to UBA-52582, Table 1).

The ^14^C results demonstrates that there is an increase in the number of reindeer from *c.* 11,600 cal BCE to *c.* 10,600 cal BCE, where there is a significant drop in the abundance of reindeer, representing the beginning of the Younger Dryas cold period. Then there is slow increase in reindeer abundance until *c.* 9200 cal BCE where reindeer slowly starts to be less abundant until the latest dated reindeer from Skåne around *c.*7200 cal BCE (Figure 2). These ^14^C results demonstrate that there has been a fluctuation in the abundance of reindeer throughout the Late Palaeolithic and Early Mesolithic.

We have used the median values of each calibrated radiocarbon date in the figures and discussion, for all values see Table 1.

### Habitat, stable δ^13^C and δ^15^N isotopes

We measured δ^13^C and δ^15^N isotopes in unburnt reindeer skeletal remains. In total 174 samples (antlers *n* = 132, bones *n* = 41 and teeth *n* = 1) gave collagen that fulfilled the quality criteria for well-preserved collagen (Ambrose, 1990; DeNiro, 1985; van Klinken, 1999; Table 1 and Supplemental Table S1).

**Figure 3.**
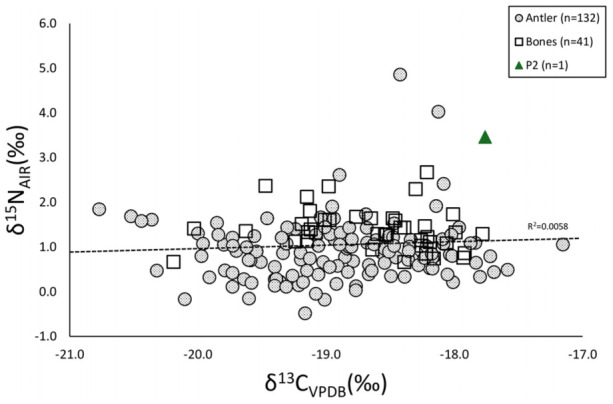
Stable δ^13^C and δ^15^N isotope results of reindeer in this study. Less than 1% (R^2^ = 0.0058) of the variation of the δ^13^C relates to the δ^15^N values.

**Figure 4.**
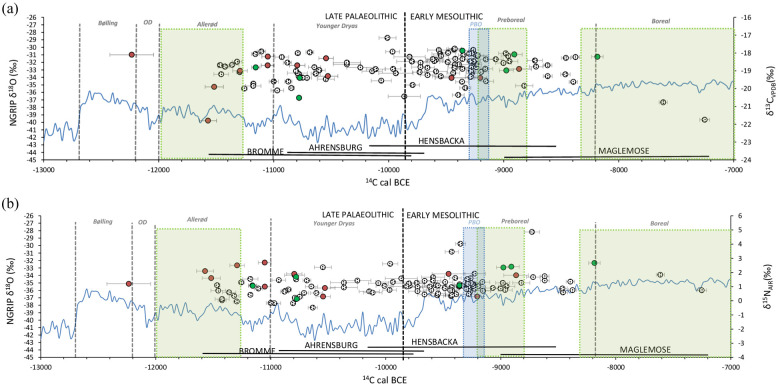
(a) Radiocarbon and δ^13^C data from reindeer in Southern Scandinavia, together with δ^18^O NGRP data from the Greenland Ice core (blue curve; Rasmussen et al., 2006). Grey circles represent reindeer from Southern Sweden, green circles represent reindeer from western Sweden, and red circles represent reindeer from Denmark (Wild et al., 2020). Radiocarbon data are presented as BCE (Before Common Era). The green boxes represent estimated periods of presence of land-bridges between continental Europe and Sweden (Björck, 1995). The blue box represents the timing of the Preboreal Oscillation event, that is, c. 9.4 BCE (Stroeven et al., 2016). (b) Radiocarbon and δ^15^N data from reindeer in Southern Scandinavia, together with δ^18^O NGRP data from the Greenland Ice core (blue curve; Rasmussen et al., 2006). Grey circles represent reindeer from Southern Sweden, green circles represent reindeer from western Sweden, and red circles represent reindeer from Denmark (Wild et al., 2020). Radiocarbon data are presented as BCE (Before Common Era). The green boxes represent estimated periods of presence of land-bridges between continental Europe and Sweden (Björck, 1995). The blue box represents the timing of the Preboreal Oscillation event, that is, c. 9.4 BCE (Stroeven et al., 2016). *Note*. Please refer to the online version of the article to view this figure in color.

The δ^13^C for all skeletal elements (*n* = 174) varies from −21.8‰ to −17.2‰ with a mean and standard deviation of −18.9‰ ± 0.7‰ (Table 1, Figure 3), that is, the values are heterogenous and vary substantially both on a local and regional level. The δ^13^C values for both antlers and bone are similar (antler; −21.8‰ to −17.2‰, mean = −19.0 ± 0.9‰, bone; −20.0‰ to −17.8‰, mean = −18.6 ± 0.5‰). The one tooth (P^2^) had a δ^13^C value of −17.8‰ (Figure 3). The large variation in the δ^13^C values can be related to changes in climate over this long timespan (Figure 4a), affecting for example, precipitation (Amundson et al., 2003) or events affecting the canopy effect (Drucker et al., 2008; Tieszen, 1991).

**Figure 5.**
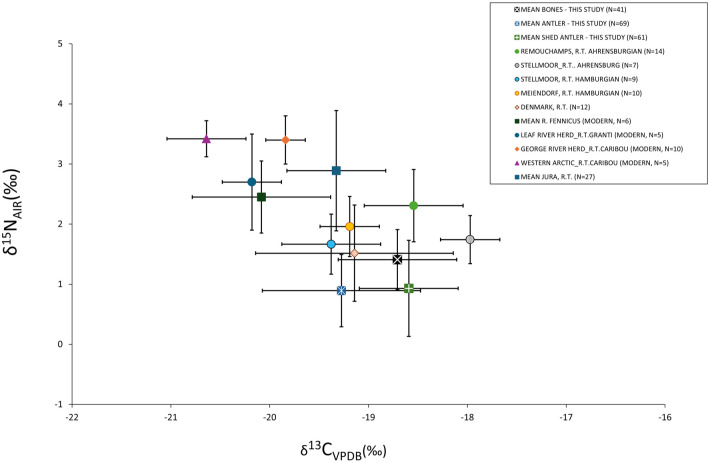
Mean values of each skeletal element for the δ^13^C and δ^15^N isotope results in this study. Added are isotopic values of palaeolithic reindeer from Denmark (bone/antler), northern Germany (bone), and the French Jura (bone; see Bridault et al., 2000; Drucker et al., 2012; Ramsey et al., 2002; Wild et al., 2020), as well as mean δ^13^C and δ^15^N isotope values for modern wild reindeer/caribou from Canada and Alaska, as well as Rangifer tarandus fennicus from northern Finland.

The δ^15^N values, in all skeletal elements (*n* = 174), varies from −0.5‰ to 4.9‰ with a mean and standard deviation of 1.0‰ ± 0.7‰. Also, for the δ^15^N values, the variation for antler and bone are different (antler; −0.5‰, 4.9, and mean = 0.9‰ ± 0.7‰, bone; 0.7, 2.7, and mean = 1.4‰ ± 0.5‰), and the mean value for the bones is slightly higher than that for the antler (~+0.5‰; Figure 5). The one tooth (*P*^2^) had a δ^15^N value of 3.5‰ (Figures 2 and 4b).

**Figure 6.**
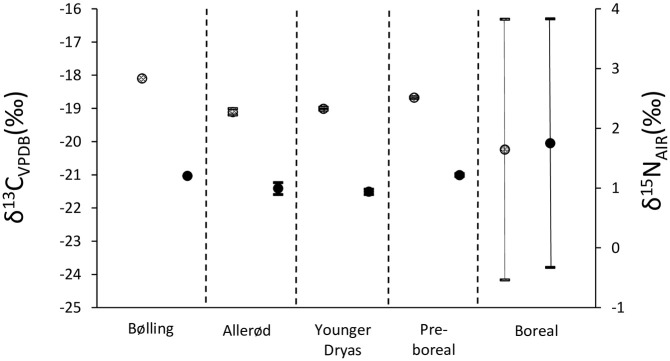
Mean stable δ^13^C and δ^15^N isotope values with 95% confidence intervals plotted against different geochronozones (filled striped circles = δ^13^C; filled black circles = δ^15^N).

In order to explore changes in relation to changing climate we plotted mean values of δ^13^C and δ^15^N for each geochronozone with confidence intervals (95%), and a *t*-test was conducted. We found significant differences between the different geochronozones. Reindeer had higher δ^13^C values at the onset of the Early Mesolithic that would indicate a warmer climate (Table 3, Figure 6).

## Discussion

### Chronology in the presence or absence of reindeer in southern Sweden

The immigration of reindeer from Denmark into southern Sweden in the Late Palaeolithic can be attributed to various factors. Previous research suggested that climate change and changing vegetation patterns drove reindeer northward, via the first land-bridge emerging between Sweden and Denmark during the Bølling-Allerød transition (Björck et al., 1996). This corresponds to the first occurrence of reindeer in southern Sweden, that is, 12,066–11,398 cal BCE (11,770 ± 140 BP, Lu-3262; Sommer et al., 2011). Our results support this scenario (Figure 4a and b), the ^14^C results further demonstrates that the number of reindeer fluctuates over time (Figure 2). We also found differences in both δ^13^C and δ^15^N mean values for reindeer from different geochronozones (Table 3, Figure 6). As the climate gets cooler from the Bølling and Allerød to the Younger Dryas geochronozones, both δ^13^C and δ^15^N mean values in reindeer decreases and the number of reindeer increase to reach its maximum during the Younger Dryas. As the climate gets warmer at the beginning of the Early Mesolithic (the Preboreal geochronozone), the δ^13^C and δ^15^N mean values increases and the number of reindeer decreases. Hence, there is a correlation between climate shifts and number of reindeer. The three reindeer from the Boreal geochronozone, has the largest diversity, indicating a changing environment into a more dense forest and, yet again, increasing temperatures. We interpret the fluctuations in reindeer as a reflection of favourable or less favourable environments for reindeer.

At the transition between Allerød and the Younger Dryas (*c*. 11,000 cal BCE; Figures 2 and 4a and b), with a change in climate towards a colder climate, we notice a stagnation in the number of radiocarbon dated reindeer samples. Then the number of reindeer increase, which can be put in connection to a favourable vegetation and climate during the colder Younger Dryas. Then, around *c.* 10,600 cal BCE (Figure 2) there is a sharp drop in the number of radiocarbon dated reindeer, after which the reindeer population slightly increases to reach a peak around 9400–9200 cal BCE. There is no land-bridge during the drop of reindeer around 10,600 cal BCE, suggesting that no new reindeer could have migrated into southern Sweden during that interval. However, we suggest that the increase in reindeer, after the drop during the Younger Dryas, was related to the more favourable environmental conditions for reindeer.

According to Björck et al. (1996) there were periods when reindeer were absent on the Scandinavian peninsula, however the ^14^C results demonstrates that reindeer were present in southern Sweden from the mid-Allerød all through to the end of the Preboreal geochronozones, although the numbers fluctuate (Figure 4a and b). During the Preboreal oscillation cold event, the number of radiocarbon dated reindeer increases (Figures 2, 4a and b). There is a gap at the onset of the early Boreal of approximately 600 years between the two latest dated reindeer from southern Sweden and the latest dated reindeer from western Sweden. This suggest that reindeer had disappeared from southern Sweden by this point, and that the two reindeer from southern Sweden dating to the Boreal geochronozone were either late migrants from Denmark or formed part of a local population that were still present in southern Sweden.

In southern Norway and western Sweden, it is not until *c*. 8000–7000 cal BCE that the deglaciation of the Fennoscandian Ice Sheet has reach far enough in order for animals to graze (Hughes et al., 2016: 31). This suggests that reindeer probably were not, or at least not without crossing larger areas of ice cover, able to cross the Oslofjord until that period. Jonsson (2018) has a similar argument for western Sweden adding on the strong current in the straits of the Göta Valley and Uddevalla (p. 19, 25). Though, it has been suggested that humans were present in Norway *c.* 8200 cal BCE (Lie, 1990: 12,15), the ^14^C results of reindeer skeletal remains from western Sweden, demonstrate that reindeer were present in western Sweden prior to any known human settlement sites. Reindeer is present in western Sweden during three periods: 11,157–10,774 cal BCE (*n* = 3), 9359–8908 cal BCE (*n* = 3) and around 8186 cal BCE (*n* = 1; Figure 4a and b). These are periods that either post-dates the introduction of reindeer or the increase of reindeer in southernmost Sweden, and since the vegetation in western Sweden is similar to southern Sweden, that is, favourable to reindeer, we cannot completely disregard the fact that reindeer might have moved northwards following favourable habitats into western Sweden. According to Hughes et al. (2016) and Mangerud et al. (2016) an ice sheet would have blocked any passages between the Oslofjord and Sweden until *c*. 9000 cal BCE, suggesting that reindeer present in western Sweden would have come from the west after that period. An additional scenario, suggested by Bang-Andersen (2003) is that reindeer might have migrated into southern Norway from southern Sweden on the winter ice.

In south-eastern Sweden reindeer could have thrived at least until the Late Preboreal, since the vegetation was favourable (Blaesild et al., 2024). After this, less favourable vegetation is introduced. However, there is only one ^14^C-dated reindeer (9319–8760 cal BCE) from the area (Hallgren, 2018) indicating very low abundance of the species. This ^14^C dating corresponds to the Preboreal Oscillation and the creation of a land-bridge at the Öresund strait, making it possible for new reindeer to enter. One hypothesis that might explain the lack of reindeer in south-eastern Sweden is the growing competition from more temperate-adapted species, such as moose and red deer. For instance, at Dagsmosse in southeastern Sweden a multitude of land-living mammals are represented (Gummesson, 2019), except for reindeer. Taphonomic reasons, such as chemical processes in the soil affecting bone preservation, or glacial and land movement destroying or moving the reindeer remains can be dismissed as the faunal assemblage contains other species.

### Reindeer habitat in the Late Palaeolithic and Early Mesolithic

The δ^13^C values of the reindeer from Southern Sweden vary substantially (Figures 4a, b and 5). In contrast, except for a few outliers, there is little variation over time in δ^15^N isotope values (Figure 4b) and there is no correlation between the two isotopes (*R*^2^ = 0.0058; Figure 3).

A decrease in δ^13^C may indicate environmental shift (Drucker et al., 2012: 325). An increase in more varied and dense forests during the Boreal geochronozone would affect δ^13^C isotope values for example, by a canopy effect (Drucker et al., 2008). We observe systematic chronological changes in δ^13^C values in reindeer with higher δ^13^C values at the onset of the Early Mesolithic. The variation in δ^13^C isotope values in reindeer in southern Sweden might also be due to a reduced or increased lichen availability, again caused by shifts in the environment or that the reindeer grazed in different areas (Figure 4a). However, this needs further investigation.

Additionally, reindeer from the Late Palaeolithic and the Early Mesolithic from Denmark, that is, dated to the same period to reindeer in this study (Figure 4a and b), have similar δ^13^C and δ^15^N values (Wild et al., 2020). Reindeer were simultaneously present in Denmark and Sweden when there was no land-bridge between the areas, thus reindeer (Figure 4a and b) were isolated from each other after the first land-bridge, grazing in different areas and representing different herds. Although we know that Canadian caribou populations can cross sea ice up to distances of several kilometres (Poole et al., 2010), which might also have been a possibility for south Scandinavian reindeer, if the ice situation would have allowed them to cross. From vegetation data (e.g. pollen), the landscape has been described as a steppe tundra (Larsson, 1994) with presence of both *Betula* and *Pinus* (Table 2), a landscape and vegetation reindeer is well adapted to. It isn’t until the Boreal chronozone with the introduction and increase of more dense forests including species such as *Ulmus* and *Quercus* (Table 2) that the abundance of reindeer decreases. The two youngest reindeer from southern Sweden, that lived during the Boreal geochronozone, consequently have the lowest δ^13^C values which might be due to a canopy effect caused by the implementation of a denser forest including more varied species (Figure 4a). The number of reindeer decreased dramatically during this period, thus the change in vegetation and climate might have caused reindeer to finally disappear from southern Scandinavia. Further and most important, based on the lack of reindeer remains north of southern Sweden, it is evident that reindeer did not follow the retreat of the Fennoscandian Ice Sheet.

**Table 2.** Overview of Late Palaeolithic and Early Mesolithic vegetation in southern, western north-central Sweden (Antonsson et al., 2006; Antonsson and Seppä, 2007; Björck et al., 2001) as well as in Östergötland, Sweden (Blaesild et al., 2024) and western Norway (Birks, 1994).

^14^C (BCE)	Östergötland (Blaesild et al., 2024)	Southern Sweden (Björck et al., 2001)	Western Sweden (Trehörningen; Antonsson and Seppä, 2007)	North-Central Sweden (Gilltjärnen; Antonsson et al., 2006)	Western Norway (Birks, 1994)	Geochronozones
>9500			Betula (+), Hippophäe rhamnoides (−)	Betula, Pinus	Salix, Betula	Preboreal
*c*. 9500–9100	Betula, Juniperus, B. nana, Rubus chamaemorus, Vaccinum and Calluna vulgaris	Pinus, Betula	Betula, Pinus, Corylus, Salix	Betula, Pinus, Corylus		
*c*. 9100–8500	Betula (+), Pinus (−), Juniperus (−), Betula nana (−), Artemisia (−), Rubus chamaemorus (−)	Betula, Artemisia, Pinus, Poceae, Chenopodiaceaae	Betula, Pinus, Corylus, Salix	Corylus	Salix, Betula (−), Artemisia, Sedum	
*c*. 8500–8200	Pinus (−), Betula (+), Corylus, Ulmus, Alnus (−)	Empetrum, Betula, Pinus, Pocaceae				
*c*. 8200–7800	Pinus (−), Betula (+), Corylus, Ulmus, Alnus (−)	Juniperus, Betula, Salix	Corylus, Ulmus, Alnus, Quercus, Fraxinus	Alnus, Betula, Pinus	Empetrum (+), Betula (+)	Boreal
*c*. 7800–7150	Corylus (+), Pinus (−), Betula (+), Artemisia, Pteridium, Alnus, Ulmus, Quercus, Fraxinus	Betula, Pinus, Corylus				

We know that different modern sub species of reindeer are adapted to different environments, where for instance, *Rangifer tarandus fennicus* are more adapted to Boreal conditions than the tundra reindeer. Also, according to Syroječkovskij (1995: 151) there is a small competition between groups of tundra and forest reindeer, especially if they were to feed on limited lichen resources. This suggests the possibility of reindeer populations of different phenotypes crossing the land-bridge. According to Björck et al. (1996) approximately 76% of the reindeer antlers found in southern Sweden are from *Rangifer tarandus* and 24% from *Rangifer tarandus fennicus*, mainly based on size differences of the antlers (p. 195). Further aDNA analysis on reindeer could elucidate this issue. However, forest reindeer might not have been one of the pioneer reindeer to disperse into Sweden since the early post-glacial environment in Southern Sweden was not their ecological preference. Björck et al. (1996) also argued that a land-bridge along with climate, vegetation, and palaeogeographical factors are interconnected factors important for dispersal of reindeer and cannot be isolated from each other (p. 210). Kellner et al. (2024), using mtDNA of reindeer from Svalbard, before and after human colonisation of the island, demonstrated that hunting resulted in major genetic changes and reconstruction in reindeer populations. Hence, genetic studies of the southern and western Swedish reindeer populations could contribute to the issue and on whether reindeer were hunted.

Differences in the δ^15^N values is caused by biochemical changes, changes in precipitation, temperature, altitude, soil composition as well as starvation that might affect nitrogen uptake for different reindeer (Amundson et al., 2003; Barboza and Parker, 2006; Drucker et al., 2003, 2012; Nieminen and Pietilä, 1999; Parker et al., 2005; Schwartz-Narbonne et al., 2021; Stevens et al., 2008). Reindeer from different periods and contexts have different δ^13^C and δ^15^N isotopic values (Figure 5). For instance, reindeer from the Upper Palaeolithic in France and Germany have, in general, increased δ^15^N isotope values (Drucker et al., 2012) compared to reindeer in this study (Figure 5), just as modern wild reindeer (*Granti* and *Caribou*) from Northern America and northern Finland (*Fennicus*) that have increased values in both δ^13^C and δ^15^N (Britton, 2010; Fjellström et al., 2023) compared to the values in this study. This suggests that the isotope values reflect differences in habitat and behaviour. Contemporary reindeer also have varying δ^15^N isotope values, suggesting that these reindeer grazed in different areas. According to both Stevens et al. (2008) and Drucker et al. (2012), there is a correlation in reindeer with the low δ^15^N values and specific permafrost ecosystems, where low nitrogen content in soil and plants is a consequence of specific ecosystems (p. 43). It has also been suggested that low δ^15^N values in Late Palaeolithic/Early Mesolithic reindeer reflect a cooling climate and also wetter conditions (Stevens et al., 2008: 42), whereas increased δ^15^N values would indicate hot/arid temperature (Amundson et al., 2003). Increased δ^15^N values on the other hand has been recorded as being caused by seasonal stress in contemporary cervid bone collagen (Schwartz-Narbonne et al., 2021). There are two reindeer with increased δ^15^N values (>3.5‰) both dated to the Preboreal Oscillation, also referred to as the 11.4 BP event (Figure 4b). The high value from one tooth (P_2_) from Hässleberga could be due to a weaning effect. The other increased value come from a reindeer from Dagstorps mosse and is dated to a period of a cold climate. Could the increased δ^15^N isotope value of this antler, that represents a few months of the year of the reindeer’s life, illustrate a shorter warmer stress period within the 11.4 BP event? There are a few outliers in both the δ^13^C and δ^15^N values that could be explained by a shift in climate and/or habitat (Figure 5).

We find statistically significant non-overlapping 95% confidence intervals for both δ^13^C and δ^15^N mean values between different geochronozones, where Allerød and Younger Dryas, that has overlapping confidence intervals, differ from the Preboreal geochronozone. The Boreal geochronozone confidence interval, encompasses all other geochronozones (Table 3, Figure 6). The mean values between the different geochronozes increases for both δ^13^C and δ^15^N from the Late Palaeolithic to the Early Mesolithic in accordance with a change towards a warmer climate in the Preboreal geochronozone.

**Table 3.** T-test of mean δ^13^C and δ^15^N values for different geochronozones with a 95% confidence interval.

Geochronozones	δ^13^C (‰)	SD (‰)	δ^15^N (‰)	SD (‰)	N	Low δ^13^C (‰)	High δ^13^C (‰)	Diff	Low δ^15^N (‰)	High δ^15^N (‰)	Diff
Bølling	−18.1	-	1.2	-	1			n/a			n/a
Allerød	−19.1	0.9	1.0	0.8	19	−19.2	−19.0	a	0.9	1.1	a
Younger Dryas	−19.0	0.7	0.9	0.7	33	−19.1	−19.0	a	0.9	1.0	a
Preboreal	−18.7	0.6	1.2	0.8	70	−18.7	−18.7	b	1.2	1.2	b
Boreal	−20.2	1.8	1.7	1.0	3	−24.2	−16.3	ab	−0.3	3.8	ab

“The letters ‘a’ and ‘b’ indicate groups that are statistically different. Groups that share the same letter do not differ significantly, and a group marked ‘ab’ does not differ significantly from either ‘a’ or ‘b’.”

### Human-reindeer interactions

Riede (2014b) suggests that the Laacher See volcanic eruption *c.* 11,000 cal BCE would have caused human mobility in the late Allerød, in this he also includes mobility of terrestrial fauna, such as reindeer. It is known from modern caribou (*Rangifer tarandus granti* and *groenlandicus*) populations in Alaska and Canada that they migrate annually between 800 and 5055 km (see Galán López et al., 2023). As the crow flies, it is *c.* 660 km from the Laacher See to the southern tip of Sweden which is not long for reindeer mobility. Hence it is possible that northern European reindeer populations moved long distances, annually. But to what extent was reindeer incorporated into people’s mobility and everyday life and diet?

In western Sweden, reindeer presence falls within the occurrence of the Hensbacka culture (*c.* 10,200–8500 cal BCE, see Schmitt et al., 2006; Figure 4a and b). Western Sweden, specifically Central Bohuslän, has been interpreted as a central seasonal exploitation area for hunter-gatherers travelling from North-Central Europe, which eventually grow into developing a short-distance seasonal migration (Schmitt et al., 2006: 24). Referring to different theories of human mobility, Schmitt et al. (2006) discuss different strategies of how western Sweden came to be populated, of which one was directly via the Öresund strait, and another to southern Norway via Doggerland (p. 23). These strategies of mobilities could be applied to reindeer mobility as well. However, according to Svendsen (2018: 369), the need for people might have been different, as coastal environments in Norway were resourceful and well adapted to the need of the first pioneers, as compared to the inland and the mountains for eventual reindeer hunting.

Little is known about the diet of the first human colonists, however, it has been demonstrated that Early Mesolithic human population’s diet varied considerably (see figure 2 in Eriksson and Lidén, 2025). A female human from Österöd in coastal western Sweden, ^14^C-dated to 8274–7935 cal BCE (Ahlström and Sjögren, 2007: 56), had a diet composed mostly of marine protein, just as the human individuals from Huseby Klev that were highly dependent on marine subsistence strategies (Lidén et al., 2004: 26; Boëthius, 2018: 122). Even though reindeer was present, there is no indication that reindeer was consumed to any larger extent during the Early Mesolithic. The only exception for reindeer consumption are a few modified reindeer skeletal remains from kettle holes in Hässleberga (Magnell et al., 1999). There is little evidence in Sweden of any worked, modified or refitted reindeer skeletal remains found in archaeological contexts in the earliest Stone Age. According to Wild et al. (2020: 130) the reindeer antler is a preferred raw material among the pioneer colonisers of Denmark; however, a shed antler does not mean hunting. Additionally, Larsson (1991) has suggested that the interpretation of the first colonisers as “reindeer hunters” has been exaggerated (pp. 11–12). His argumentation is partly based on the fact that finds of elk exceed those of reindeer at Bromme in Denmark, and that little attention has been paid to other species also present and introduced during the same time. This suggests that reindeer was, probably not, the most important food resource for the early colonisers of the Scandinavian peninsula. We also suggest that there is too little evidence of human-reindeer relations in order to argue that the first pioneers in southern Sweden were reindeer hunters, and rather that reindeer played some role in the general subsistence strategy.

The abrupt decrease in reindeer population size and absence in the archaeological and fauna records raises questions about its causes. Can it be linked to a change in the use of reindeer – as burnt skeletal remains in hearths might indicate? Instances of cremated reindeer skeletal remains are common in northern Sweden. The presence of cremated reindeer in Dalarna during the early Boreal period (Wehlin, 2014) could eventually give a hint to a change in subsistence patterns dealing with reindeer. In contrast to the idea that there is a shift from hunting reindeer towards a more marine subsistence, archaeological remains hints towards that reindeer skeletal remains might appear in other forms, here as cremated bones from hearths. According to Ekholm (2021), reindeer entered central and northern Sweden from the northeast and the south (p. 90). Exploring the use of reindeer skeletal remains for fuel, food, and raw materials is crucial for a more comprehensive understanding of reindeer dispersal in the Early Holocene. Additional δ^34^S (see Stevens et al., 2025), δ^18^O_p_ and ^87^Sr/^86^Sr analysis, as well as aDNA analysis can help us better understand reindeer dispersal between Southern Norway and Sweden. Or could it be other natural causes of the disappearance of reindeer? In a study on caribou from northeast Canada (Le Corre et al., 2016: 269), arrival to calving grounds is later when winters has been mild and precipitation been abundant during migration. Shifts in climate has an effect on caribou migration, which could also have been the case for reindeer from southern Sweden. The greater the herd size is the less time they spent on their calving grounds, probably due to limited grazing, reflected in their diet. Another consequence on less time spent on calving grounds due to climatic shifts and larger herds, might be reduced calving. This would all in all reduce reindeer population sizes and eventually lead to extinction.

## Conclusions

This study contributes to debates surrounding the postglacial expansion of reindeer into southern Sweden. Reindeer first arrived during the Allerød when there was a land-bridge between Denmark and Sweden (*c.* 12,000–11,300 cal BCE), then when there is no land bridge and reindeer in Sweden are isolated. The abundance of reindeer varies and reaches a peak just before the Preboreal oscillation (*c*. 9300–9150 cal BCE). We also show that reindeer were present in western Sweden during three phases, and that they were probably introduced from the south, but migration from southern Norway cannot be entirely dismissed. We suggest that reindeer were present in south-eastern Sweden during times when the environment was favourable.

Changes in the δ^13^C and δ^15^N values in reindeer shifts in accordance with shifts in the climate and environment. As the climate gets cooler with a favourable reindeer habitat in the Late Palaeolithic, δ^13^C and δ^15^N isotope values decreases and the number of reindeer increases. In contrast, the number of reindeer decreases with increasing temperatures in the Early Mesolithic corresponding to the Preboreal and Boreal geochronozones. Increases in δ^13^C and δ^15^N values by the end of the Preboreal and the Boreal geochronozone periods with and increasing and denser forest, indicate an unfavourable habitat for reindeer.

Lack of evidence for human-reindeer interactions indicate that reindeer was not a main subsistence for the human pioneers but rather one element of their subsistence. Accordingly, the extinction of reindeer in southern Scandinavia was probably a cause of several climatic and environmental factors, rather than extirpation of the human pioneers.

## Supplemental Material

sj-xlsx-1-hol-10.1177_09596836261422209 – Supplemental material for Tracing the early dispersal of reindeer in southern Sweden: Chronology, habitat, and human interaction (c. 12,000–7000 BCE) Supplemental material, sj-xlsx-1-hol-10.1177_09596836261422209 for Tracing the early dispersal of reindeer in southern Sweden: Chronology, habitat, and human interaction (c. 12,000–7000 BCE) by Markus Fjellström, Peter Jordan, Anders Angerbjörn, Anna-Kaisa Salmi and Kerstin Lidén in The Holocene

sj-xlsx-2-hol-10.1177_09596836261422209 – Supplemental material for Tracing the early dispersal of reindeer in southern Sweden: Chronology, habitat, and human interaction (c. 12,000–7000 BCE) Supplemental material, sj-xlsx-2-hol-10.1177_09596836261422209 for Tracing the early dispersal of reindeer in southern Sweden: Chronology, habitat, and human interaction (c. 12,000–7000 BCE) by Markus Fjellström, Peter Jordan, Anders Angerbjörn, Anna-Kaisa Salmi and Kerstin Lidén in The Holocene
